# Epigenetic modification of mesenchymal stromal cells enhances their suppressive effects on the Th17 responses of cells from rheumatoid arthritis patients

**DOI:** 10.1186/s13287-018-0948-4

**Published:** 2018-08-09

**Authors:** Kyoung-Woon Kim, Hye Joung Kim, Bo-Mi Kim, Yong-Rim Kwon, Hae-Rim Kim, Yoo-Jin Kim

**Affiliations:** 1 Convergent Research Consortium for Immunologic Disease, Transplant Research Center, The Catholic University of Korea, Seoul, Republic of Korea; 2 Laboratory of Hematological Disease and Transplant Immunology, The Catholic University of Korea, Seoul, Republic of Korea; 3 Department of Internal Medicine, Konkuk University School of Medicine, Seoul, Republic of Korea; 4 Seoul St. Mary’s Hematology Hospital, College of Medicine, The Catholic University of Korea, 222 Banpo-daero, Seocho-Gu, Seoul, 06591 Republic of Korea

**Keywords:** Th17 cells, Human mesenchymal stromal cells, Epigenetic modification, Rheumatoid arthritis

## Abstract

**Background:**

The aim of this study was to investigate if epigenetically modified human mesenchymal stromal cells (hMSCs) can regulate the Th17-related immune responses.

**Methods:**

We tested epigenetic drug combinations at various doses and selected the four combinations that resulted in maximal interleukin (IL)-10 and indoleamine 2,3-dioxygenase gene expression in hMSCs. We examined the effects of epigenetically modified hMSCs (epi-hMSCs) on CD4^+^ T-cell proliferation and inflammatory cytokine secretion under Th0- and Th17-polarizing conditions using mixed lymphocyte reactions and enzyme-linked immunosorbent assays (ELISAs). We determined Th17 cytokine levels and the percentage of Th17 cells among synovial fluid mononuclear cells (SFMCs) from rheumatoid arthritis (RA) patients by ELISA and flow cytometry.

**Results:**

Epi-hMSCs inhibited the development of IL-17-producing cells in culture. The percentages of IL-17^+^ and interferon (IFN)-γ^+^ cells among peripheral blood mononuclear cells from healthy donors were lower under both the Th0 and Th17 conditions in the presence of epi-hMSCs than in the presence of no or untreated hMSCs. Epi-hMSC-treated RA patient SFMCs secreted lower levels of IL-17 and IFN-γ than RA patient SFMCs cultured without hMSCs or with untreated hMSCs.

**Conclusions:**

An optimal combination of hypomethylating agents and histone deacetylase inhibitors can enhance the immunomodulatory potential of hMSCs, which may be useful for RA treatment.

## Background

Rheumatoid arthritis (RA) is a chronic inflammatory disease characterized by uncontrolled synovitis and subsequent destruction of the cartilage and bone. In RA, interleukin (IL)-17 induces the production of proinflammatory mediators, such as IL-1 and tumor necrosis factor (TNF)-α, from synovial fibroblasts, macrophages, and chondrocytes. The levels of IL-17 produced by CD4^+^ T cells in the synovium are significantly higher in patients with RA than in patients with osteoarthritis [1, 2]. T-helper (Th)17 cells, which are defined by their selective IL-17 secretion, are a distinct lineage of CD4^+^ helper T cells. Their development and activity are regulated by Th1 and Th2 cytokines [3–5].

Mesenchymal stromal cells (MSCs) are present in various tissues, including the bone marrow (BM), umbilical cord blood, and adipose tissues. They possess intrinsic immunoregulatory properties that modulate innate and adaptive immunity [6–9]. As a result, MSCs have been widely studied as a promising platform for a cell-based therapy to prevent or treat RA. The proof of principle has been established in preclinical studies [10–14]. BM-MSCs can suppress T-cell proliferation via contact-dependent mechanisms and produce soluble immunoregulatory factors, such as IL-10 [15]. The immune-altering abilities of MSCs have been exploited for therapeutic purposes, as in the ongoing clinical trials of the transfer of MSCs for the treatment of rheumatic diseases [12].

DNA methyltransferases and histone deacetylases (HDAC) are epigenetic modulators that regulate DNA methylation and the histone acetylation status of genomic DNA, respectively, thereby regulating the expression of target genes. Different cell types possess different methylation and histone status profiles. Responses to hypomethylating agents (HMAs) or HDAC inhibitors (HDACi) vary according to individual cellular characteristics. HMAs and HDACi increase the efficiency of induced pluripotent stem cell generation and somatic cell reprogramming through epigenetic modifications [16–19]. Recent studies showed that an HMA mitigated graft-versus-host disease and experimental colitis by converting effector T cells to regulatory T cells [20–23]. In our previous study, epigenetic modification of human MSCs (hMSCs) with HMAs plus HDACi resulted in higher expression of Runx-2, BDNF, and Sox-9 than the control treatment. HMAs and HDACi enhance the in-vitro differentiation of MSCs, suggesting that epigenetic modification could alter the function of hMSCs [22].

In this study, we aimed to investigate if epigenetic modification could enhance the immunoregulatory properties of hMSCs, enabling them to inhibit the production of proinflammatory cytokines by T cells and suppress Th17 responses in cells from patients with RA.

## Methods

### Patient populations and study design

We obtained synovial fluid from six RA patients, all of whom fulfilled the 1987 revised criteria of the American College of Rheumatology (formerly the American Rheumatism Association) [24]. We also obtained peripheral blood from six healthy volunteers.

### Culture and assessment of MSCs

We acquired hMSCs, which were isolated and expanded from healthy donor BM, from the Catholic Institute of Cell Therapy (Catholic Medical Center, Seoul, Republic of Korea). We expanded hMSCs up to passage 3. After the third passage, we assessed the cells for the MSC phenotype CD73^+^CD90^+^CD105^+^CD11^−^CD19^−^CD34^−^CD44^−^HLA-DR^−^ using APC-conjugated anti-CD73, FITC-conjugated anti-CD90, PerCP 5.5-conjugated anti-CD105, APC-conjugated anti-CD11, PE-conjugated anti-CD19, FITC-conjugated anti-CD34, PerCP 5.5-conjugated anti-CD45, and FITC-conjugated anti-HLADR. For the phenotypic analysis of MSCs, each sample was divided into three aliquots to prevent the occurrence of interference waves. All antibodies were purchased from BD Biosciences (San Diego, CA, USA). We determined the level of nonantigen-specific fluorescence by incubating cell aliquots with isotype-matched monoclonal control antibodies. We analyzed the samples with a FACSCalibur™ flow cytometer (BD Biosciences, San Diego, CA, USA) using FACSDiva™ software. For each analysis, we assayed a minimum of 10,000 cells.

### Combination treatment with HMAs and HDACi

We treated hMSCs (passage 3) with a combination of HMA (5-azacitidine (5-AZA) or 5-aza-2′-deoxycytidine (DEC)) and HDACi (trichostatin A (TSA) or valproic acid (VPA)) at various doses. We used the following doses of reagents: 0.5, 1, or 2 μM 5-AZA; 50, 100, or 500 nM DEC; 50, 100, or 500 nM TSA; and 1, 5, or 10 mM VPA. In total, 36 treatment combinations were tested and the expression levels of indoleamine 2,3-dioxygenase (IDO) and IL-10 in hMSCs treated for 72 h were used to select the optimal drug combinations. All drugs were purchased from Sigma Aldrich (St. Louis, MO, USA). Real-time polymerase chain reaction (PCR) was first used to screen optimal drug combinations to maximize the mRNA expression of the genes using the following primers: IDO (*IDO1*), 5′-ACA GCG CCT TGC ACG TCT A-3′ (sense) and 5′-GAC CTT ACG GAC ATC TCC AT-3′ (antisense); *IL10*, 5′-CCA AGC CTT GTC TGA GAT GA-3′ (sense) and 5′-TGA GGG TCT TCA GGT TCT CC-3′ (antisense); and β-actin (*ACTB*), 5′-GGA CTT CGA GCA AGA GAT GG-3′ (sense) and 5′-TGT GTT GGG GTA CAG GTC TTTG-3′ (antisense). The housekeeping gene β-actin was used to normalize the gene signals. For the drug combinations selected on the basis of the mRNA levels, Western blotting was used to verify their influences on the protein level. The IDO (Santa Cruz Biotechnology, Oregon, USA), IL-10 (Santa Cruz Biotechnology), and β-actin (Santa Cruz Biotechnology) antibodies were used for this. In addition, we measured annexin V and propidium iodide staining by flow cytometry to assess cell viability after combination treatment.

### Isolation of peripheral blood mononuclear cells and synovial fluid mononuclear cells

We prepared peripheral blood mononuclear cells (PBMCs) from heparinized blood and synovial fluid mononuclear cells (SFMCs) from heparinized synovial fluids by Ficoll–Paque (GE Healthcare Life Sciences, Little Chalfont, UK) density-gradient centrifugation. Cells were cultured as described previously [25]. Briefly, we prepared cell suspensions (10^6^ cells/ml) in RPMI 1640 medium supplemented with 10% fetal calf serum (FCS), 100 U/ml penicillin, 100 mg/ml streptomycin, and 2 mM l-glutamine. We dispensed 1-ml aliquots of the suspensions into 24-well plates (Nunc, Roskilde, Denmark) for incubation. For cytokine detection at the single-cell level by flow cytometric analysis, we stimulated PBMCs with 50 ng/ml phorbol 12-myristate 13-acetate and 1 μg/ml ionomycin in the presence of GolgiStop™ (BD Biosciences) for 4 h.

### Th17-polarizing conditions

We incubated the PBMCs or SFMCs (1 × 10^6^/1 ml) from healthy individuals or patients with RA, respectively, under appropriate conditions for 48 h. To induce Th17 differentiation, we cultured PBMCs and SFMCs (1 × 10^6^/1 ml) for 48 h with anti-CD3 (1 μg/ml; BD Biosciences), anti-CD28 (1 μg/ml; BD Biosciences), IL-1β (20 ng/ml; R&D Systems, Minneapolis, MN, USA), IL-6 (20 ng/ml; R&D Systems), IL-23 (20 ng/ml; R&D Systems), and neutralizing antibodies against interferon-gamma (IFN-γ; 2 μg/ml; R&D Systems) and IL-4 (2 μg/ml; R&D Systems). To examine the immunosuppressive effects of epigenetically modified hMSCs (epi-hMSCs), we cocultured PBMCs with epi-hMSCs for 48 h and simultaneously stimulated them as described.

### CD4^+^ T-cell isolation by magnetic-activated cell sorting (MACS)

Anti-CD4 microbeads were used according to the recommendations of the manufacturer (Miltenyi Biotec, Sunnyvale, CA, USA). PBMCs were resuspended in 80 μl FCS staining buffer. Anti-CD4 microbeads (20 μl) were added and incubated for 15 min at 6–12 °C. Saturating amounts of fluorochrome-conjugated antibodies were added, and cells were incubated for an additional 10 min. Cells were diluted in 2.5 ml 2% FCS staining buffer, pelleted, resuspended in 500 μl FCS, and magnetically separated, usually on an AutoMACS magnet (Miltenyi Biotec, Bergisch Gladbach, Germany) fitted with a MACS mass spectrophotometry column. Flow-through and two 1-ml washes were collected as the negative fraction. After removal from the magnet, enriched cells were collected in two 0.5-ml aliquots from the column. Alternatively, cells stained with PE-conjugated anti-CD4 were washed, magnetically labeled with anti-PE microbeads (20 μl added to an 80 μl cell suspension for 15 min at 6–12 °C), and magnetically separated as described above. The purity of cells was assessed by flow cytometric analysis of stained cells on a FACS Vantage sorter (BD Biosciences). Most of the isolated cells (> 97%) exhibited the CD4 T-cell marker.

### Suppression assay

PBMCs were collected from healthy donors (*n* = 3). We stimulated CD4^+^ T cells with anti-CD3 (1 μg/ml) and T cell-depleted, irradiated (3000 rad) antigen-presenting cells (non-CD4^+^ T cells) in the presence or absence of epi-hMSCs. We used effector T and antigen-presenting cells (non-CD4^+^ T cells) from the same donor. The purities of the T-cell subsets were > 95% as determined by flow cytometry (data not shown). We examined the proliferation of the CD4^+^ T cells by adding [^3^H]-thymidine (1 mCi/well; GE Healthcare) to the cultures for the final 8 h of incubation. We assessed [^3^H]-thymidine incorporation using a liquid β-scintillation counter (Beckman Coulter, Brea, CA, USA). We also confirmed the immunomodulatory effect of MSCs by analyzing the epigenetic reprogramming effects on the T-cell activation/proliferation during Th17 differentiation using carboxyfluorescein succinimidyl ester (CFSE; Sigma Aldrich) labelling.

### Flow cytometric analysis of T cells

We performed flow cytometric analysis of the cells within a few hours of peripheral blood collection. We performed cell surface staining with the fluorescently labeled monoclonal antibodies anti-CD4-PE/Cy7 (RPA-T4, IgG1; BioLegend, San Diego, CA, USA) and anti-CD25-APC (M-A251, IgG1, κ; BD Biosciences). For intracellular staining, we washed the cells, then fixed, permeabilized, and incubated with the monoclonal antibodies: anti-IL-17-PE (eBio64dec17, IgG1, κ; eBioscience, San Diego, CA, USA), anti-IFN-γ-FITC (4S.B3, IgG1, κ; eBioscience), and anti-Foxp3-FITC (PCH101, IgG2a, κ; eBioscience). We also prepared appropriate isotype staining controls. We analyzed the cells using a FACSCalibur™ flow cytometer (BD Biosciences). The data were analyzed using FlowJo software (Tree Star, Ashland, OR, USA).

### Enzyme-linked immunosorbent assays

We measured the levels of cytokines, such as IL-17, IFN-γ, IL-10, and IL-2, in the CD4^+^ T-cell culture supernatants using sandwich enzyme-linked immunosorbent assays (ELISAs; R&D Systems) according to the manufacturer’s instructions. We measured the absorbance at 405 nm using an ELISA microplate reader (Molecular Devices, Sunnyvale, CA, USA).

### Statistical analysis

We performed statistical analyses with SPSS software (version 16.0; SPSS, Chicago, IL, USA). Continuous variables are summarized as mean ± standard deviation (SD). Categorical variables are summarized as a percentage of the group total. We performed independent *t* tests for continuous variables. We used the nonparametric Wilcoxon signed-rank test to compare T-cell proliferation, cytokine production, and gene expression among the control and treatment groups. We performed chi-squared/Fisher’s exact tests for categorical variables. A *P* value < 0.05 was considered statistically significant.

## Results

### The expression of IDO and IL-10 by epi-hMSCs

**Fig. 1:**
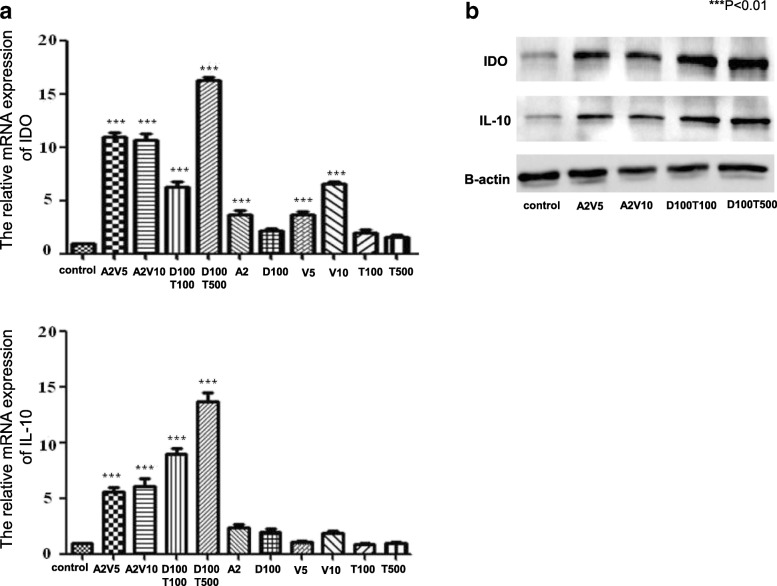
The effects of epigenetic regulators on the immunoregulatory properties of hMSCs. We quantified the expression of interleukin (IL)-10 and indoleamine 2,3-dioxygenase (IDO) mRNA in hMSCs by **a** real-time PCR and **b** Western blotting after treatment with various combinations of 5-azacitidine (A), 5-aza-2′-deoxycytidine (D), trichostatin A (T), and valproic acid (V). The data are presented as the mean ± SD, and represent three independent experiments (*n* = 3). *P < 0.05, **P < 0.01, ***P < 0.001, versus untreated control

We selected four of the 36 combinations of HMAs and HDACi based on their ability to significantly upregulate the expression of IL-10 and IDO over those in untreated hMSCs: 2 μM 5-AZA + 5 mM VPA (A2V5), 2 μM 5-AZA + 10 mM VPA (A2V10), 100 nM DEC + 100 nM TSA (D100T100), and 100 nM DEC + 500 nM TSA (D100T500). We found that the A2V10 combination had an additive effect, whereas the A2V5, D100T100, and D100T500 combinations had synergistic effects (Fig. 1a). An appreciable increase in protein expression was confirmed upon use of the four combinations selected on the basis of the gene expression results (Fig. 1b). We did not observe a higher rate of apoptosis in the drug treatment groups than in the untreated control (data not shown). Thus, the selected dosing combinations effectively increased immune regulatory molecule expression without inducing toxicity.

### The regulatory effects of epi-hMSCs on T-cell differentiation under Th0-polarizing conditions

**Fig. 2:**
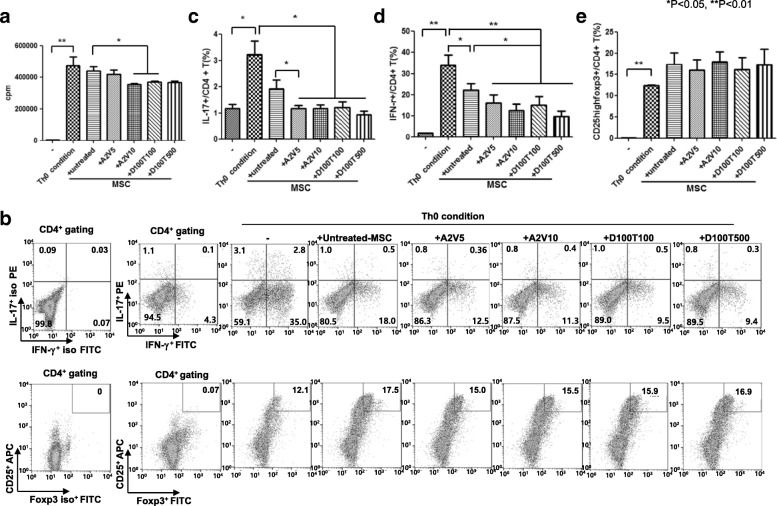
Epi-MSCs suppress the activation of T cells from healthy individuals cultured under Th0 conditions. We cocultured PBMCs from three healthy volunteers with untreated, A2V5-treated, A2V10-treated, D100T100-treated, or D100T500-treated human mesenchymal stromal cells (MSCs). Then, we cultured the cells under Th0-polarizing conditions for 48 h. **a** The proliferation of effector T cells measured using a [^3^H]-thymidine incorporation assay. PBMCs were stained with anti-CD4-PE-Cy7, anti-CD25-APC, anti-IFN-γ-FITC, anti-IL-17-PE, and anti-Foxp3-FITC. CD4^+^ T cells were gated for further analysis. **b** The percentage of CD4^+^ T cells producing IL-17, IFN-γ, and CD25^high^Foxp3^+^ was measured using flow cytometry. Then we measured the percentages of **c** IL-17^+^CD4^+^ T cells, **d** IFN-γ^+^CD4^+^ T cells, and **e** CD25^high^Foxp3^+^CD4^+^ T cells by flow cytometry (*n* = 3). The bars represent the mean ± SD. *P < 0.05, **P < 0.01. A 5-azacitidine, D 5-aza-2′-deoxycytidine, T trichostatin A, V valproic acid

PBMCs were isolated from healthy individuals and cultured in the presence or absence of epi-hMSCs under Th0 differentiation conditions. We investigated the regulatory effects of epi-hMSCs on CD4^+^ T-cell proliferation under Th0 differentiation conditions. We found that, compared with untreated MSCs, the presence of epi-hMSCs resulted in the proliferation of a smaller proportion of CD4^+^ T cells. In particular, we observed a significantly lower proportion of proliferating cells in the presence of hMSCs treated with A2V10 and D100T100 (*P < 0.05 and **P < 0.01; Fig. 2a). We also investigated the impact of epi-hMSCs on differentiation into the various T-cell subsets. The percentage of CD4^+^ T cells producing IL-17, IFN-γ, and CD25^high^Foxp3^+^ was measured using flow cytometry (Fig. 2b). The percentages of IL-17^+^CD4^+^ T cells in the cocultures with epi-hMSCs were 1.1 ± 0.2% (A2V5), 1.1 ± 0.2% (A2V10), 1.2 ± 0.4% (D100T100), and 0.9 ± 0.2% (D100T500). All four drug combinations yielded hMSCs that significantly suppressed IL-17^+^CD4^+^ T cells compared with that under the Th0 conditions alone (3.2 ± 1.0%). In addition, hMSCs treated with A2V5 significantly suppressed T-cell differentiation compared with that in the presence of untreated hMSCs (1.9 ± 0.9%; *P* < 0.05) (Fig. 2c). We also observed significantly lower percentages of IFN-γ^+^CD4^+^ T cells in cocultures with epi-hMSCs (16.2 ± 7.2%, A2V5; 12.4 ± 6.3%, A2V10; 15.1 ± 7.8%, D100T100; and 9.6 ± 5.1%, D100T500) than under Th0 conditions alone (33.9 ± 9.4%) or in the presence of untreated hMSCs (22.1 ± 6.1%) (*P < 0.05 and **P < 0.01; Fig. 2d). In contrast, coculture with epi-hMSCs resulted in a higher (though not statistically significantly) proportion of CD25^high^Foxp3^+^CD4^+^ T cells compared with culture under Th0 conditions alone (Fig. 2e).

### The regulatory effects of epi-hMSCs on Th17 cell differentiation under Th17-polarizing conditions

**Fig. 3:**
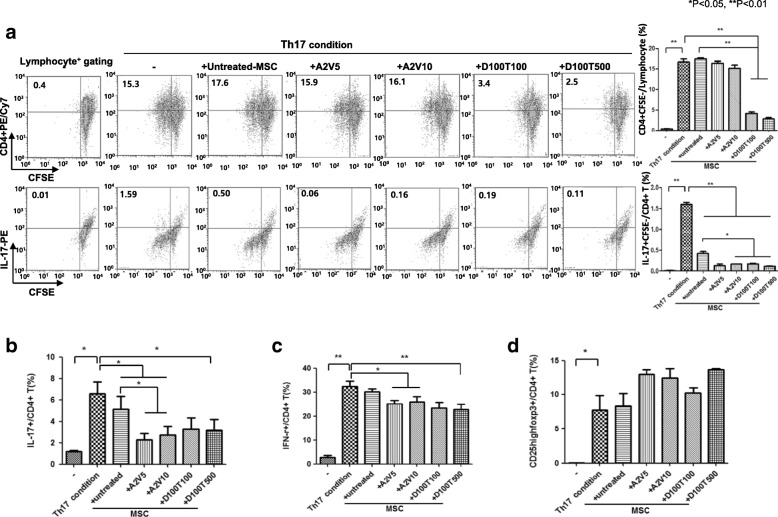
The effects of epi-MSCs on Th17 cell differentiation. We cultured human PBMCs isolated from three healthy volunteers with untreated, A2V5-treated, A2V10-treated, D100T100-treated, and D100T500-treated human mesenchymal stromal cells (MSCs). Then, we cultured the cells under Th17-polarizing conditions for 48 h. **a** The proliferation of effector Th17 cells measured using carboxyfluorescein succinimidyl ester (CFSE) staining. We measured the percentages of **b** IL-17^+^CD4^+^ T cells, **c** IFN-γ^+^CD4^+^ T cells, and **d** CD25^high^Foxp3^+^CD4^+^ T cells by flow cytometry (*n* = 3). The bars represent the mean ± SD. *P < 0.05, **P < 0.01. A 5-azacitidine, D 5-aza-2′-deoxycytidine, T trichostatin A, V valproic acid

We investigated the regulatory effects of epi-hMSCs on CD4^+^ T-cell proliferation under Th17-polarizing conditions. The effect of the immunomodulatory and regulatory functions of epi-MSCs on PBMCs was studied using a differentiation condition. The proliferation index of PBMCs was analyzed using a cell proliferation assay kit that utilized the CFSE staining method. This was further investigated by exposing PBMC-stimulated, CFSE-stained T cells to Th17 conditions and comparing the cytokine production by CFSE^dim^CD4^+^ T cells (Fig. 3a). We found that subpopulations of CFSE^dim^CD4^+^ T cells (proliferating CD4^+^ T cells) showed decreased production of the effector cytokines IL-17 under Th17 condition by MSCs with epigenetic reprogramming. Accordingly, epigenetically reprogrammed MSCs effectively inhibited Th17-specific cytokine expression (*P < 0.05 and **P < 0.01; Fig. 3a). The coculture of PBMCs isolated from three healthy donors with selected dosing combinations of epi-hMSCs significantly suppressed the proportion of Th17 cells compared with the Th17 condition (*P* < 0.05).

The percentages of IL-17^+^CD4^+^ T cells in the cocultures with epi-hMSCs were 2.2 ±1.4% (A2V5), 2.7 ± 1.9% (A2V10), 3.2 ± 2.6% (D100T100), and 3.1 ± 2.4% (D100T500), whereas those in the Th17 conditions alone and in the cocultures with untreated hMSCs were 6.5 ± 1.9% and 5.1 ± 2.4%, respectively. We found that the epi-hMSCs significantly suppressed the proportion of IL-17^+^CD4^+^ T cells in the presence of untreated hMSCs and hMSCs treated with A2V5 and A2V10 compared with those under the Th17 differentiation conditions alone (*P* < 0.05). Furthermore, we observed significantly lower proportions in the cocultures with the A2V5- and A2V10-treated epi-hMSCs than with the untreated hMSCs (*P* < 0.05; Fig. 3b). The percentages of IFN-γ^+^CD4^+^ T cells following coculture with epi-hMSCs (25.1 ± 2.8%, A2V5; 26 ± 4.3%, A2V10; and 22.7 ± 4.1%, D100T500), except for the D100T100-treated epi-hMSCs (23.4 ± 4.4%), were significantly lower than in the cultures under Th17 conditions alone (32.4 ± 4.1%). We did not observe differences in the percentages of IFN-γ^+^CD4^+^ T cells in the cocultures with epi-hMSCs and those with untreated hMSCs (Fig. 3c). In contrast, cocultures with epi-hMSCs resulted in higher proportions of CD25^high^Foxp3^+^CD4^+^ T cells (13.0 ± 1.1%, A2V5; 12.4 ± 2.5%, A2V10; 10.2 ± 1.3%, D100T100; and 13.6 ± 0.3%, D100T500) than the cultures under Th17 conditions alone (7.7 ± 4.1%) or with untreated hMSCs (8.3 ± 3.5%), although the differences were not statistically significant (Fig. 3d).

### The regulatory effects of epi-hMSCs on inflammatory cytokine production by effector T cells under Th17 differentiation conditions

**Fig. 4:**
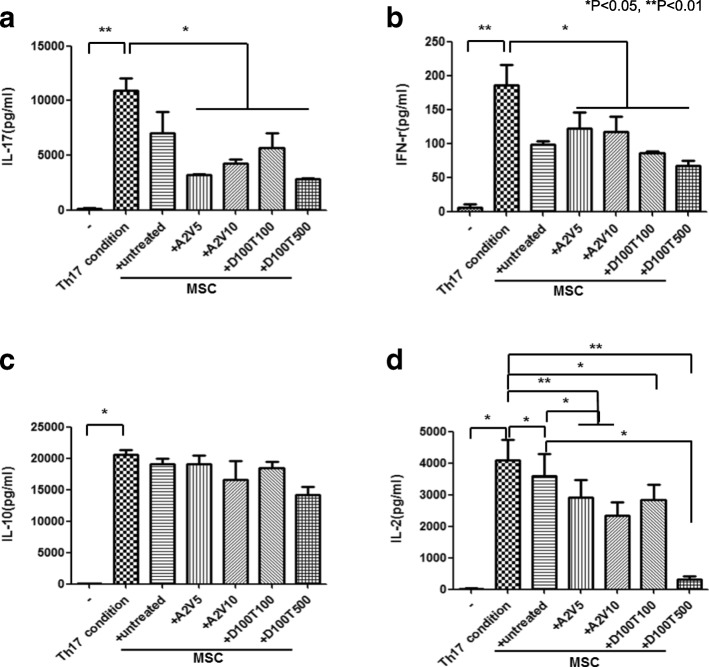
Epi-hMSCs block the production of inflammatory cytokines by activated CD4^+^ T cells. We cocultured human PBMCs isolated from three healthy volunteers with untreated, A2V5-treated, A2V10-treated, D100T100-treated, and D100T500-treated human mesenchymal stromal cells (MSCs). Then we cultured the cells under Th17-polarizing conditions for 48 h. We assessed the production of **a** IL-17, **b** IFN-γ, **c** IL-10, and **d** IL-2 in the culture supernatants by ELISA (*n* = 3). The bars represent the mean ± SD. *P < 0.05, **P < 0.01. A 5-azacitidine, D 5-aza-2′-deoxycytidine, T trichostatin A, V valproic acid

To confirm the inhibitory effects of epi-hMSCs on inflammatory cytokine production by effector T cells, PBMCs were isolated from healthy individuals and cultured in the presence or absence of epi-hMSCs under Th17 differentiation conditions. Culture supernatants under Th17 differentiation conditions resulted in significantly higher production of IL-17 (10,890 ± 2061 pg/ml), IFN-γ (185 ± 60 pg/ml), IL-10 (20,601 ± 1113 pg/ml), and IL-2 (4085 ± 1287 pg/ml) than those under nonpolarizing conditions (116 ± 227 pg/ml, IL-17; 6 ± 8 pg/ml, IFN-γ; and 21 ± 24 pg/ml, IL-2). Coculture with epi-hMSCs treated with all epigenetic drug combinations suppressed the production of IL-17, IFN-γ, and IL-2 compared with culture under Th17 conditions. In addition, we observed significantly lower IL-2 production in cocultures with epi-hMSCs than in those with untreated hMSCs, with the exception of cocultures with D100T100-treated epi-hMSCs (*P < 0.05 and **P < 0.01; Fig. 4a, b, d). However, epi-hMSCs did not affect the production of IL-10 (Fig. 4c).

### The regulatory effects of epi-hMSCs on SFMCs isolated from RA patients under Th17 cell differentiation conditions

**Fig. 5:**
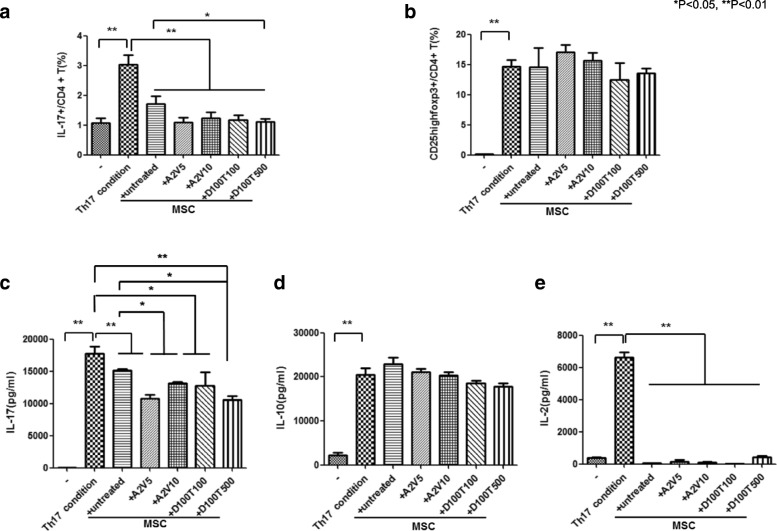
The effects of epi-MSCs on Th17-polarized CD4^+^ T cells from the SFMCs of RA patients. We cocultured SFMCs isolated from three patients with RA with untreated, A2V5-treated, A2V10-treated, D100T100-treated, and D100T500-treated human mesenchymal stromal cells (MSCs). Then we cultured the cells under Th17-polarizing conditions for 48 h. The SFMCs were stained with anti-CD4-PE-Cy7, anti-CD25-APC, anti-IL-17-PE, and anti-Foxp3-FITC. The CD4^+^ T cells were gated for further analysis of the percentage of **a** IL-17^+^CD4^+^ T cells and **b** CD25^high^Foxp3^+^CD4^+^ T cells by flow cytometry. We measured the production of **c** IL-17, **d** IL-10, and **e** IL-2 in the culture supernatants by ELISA (*n* = 3). The bars represent the mean ± SD. *P < 0.05, **P < 0.0. A 5-azacitidine, D 5-aza-2′-deoxycytidine, T trichostatin A, V valproic acid 1

We next cultured SFMCs isolated from patients with RA with epi-hMSCs. The percentages of IL-17^+^CD4^+^ T cells in the epi-hMSC cocultures were 1.1 ± 0.4% (A2V5), 1.2 ± 0.6% (A2V10), 1.1 ± 0.4% (D100T100), and 1.1 ± 0.2% (D100T500), and 3.0 ± 0.9% under Th17 conditions alone and 1.7 ± 0.7% in cocultures with untreated hMSCs (*P < 0.05 and **P < 0.01; Fig. 5a). The proportions were significantly lower in all four epi-hMSC cocultures than under the Th17 conditions alone; in addition, the proportions in the cocultures with D100T500-treated epi-hMSCs were significantly lower than those in the cultures with the untreated hMSCs. In contrast, the A2V5- and A2V10-treated epi-hMSC cocultures had higher proportions of CD25^high^Foxp3^+^CD4^+^ T cells than the cultures under the Th17 conditions alone or with the untreated hMSCs, although the differences did not reach statistical significance (Fig. 5b).

The Th17 differentiation conditions promoted significantly higher production of IL-17 (17,797 ± 2047 pg/ml) and IL-2 (6610 ± 683 pg/ml) than the nonpolarizing conditions (47 ± 75 pg/ml, IL-17; and 368 ± 112 pg/ml, IL-2). Coculture in the presence of epi-hMSCs significantly suppressed the production of IL-17 and IL-2 compared with the cultures under the Th17 conditions alone (*P < 0.05 and **P < 0.01; Fig. 5c, e). In comparison with the untreated hMSCs, the A2V5-, A2V10-, and D100T500-treated epi-hMSCs significantly suppressed IL-17 production (*P < 0.05 and **P < 0.01; Fig. 5c). However, coculture with epi-hMSCs did not affect the production of IL-10 (Fig. 5d).

## Discussion

In the present study, we observed that epi-hMSCs induced by treatment with an HMA plus an HDACi had greater immunoregulatory properties than untreated hMSCs. These findings demonstrate the potential of epi-hMSCs as a treatment option for RA.

Previous studies have reported that the immunomodulatory effects of hMSCs are associated with their abilities to inhibit Th1 and Th17 cell differentiation [26, 27]. The modulation of key markers on MSCs will be critical for developing high-performance cells for clinical application in Th17-dependent immunological diseases, such as RA. IDO is a powerful immunomodulatory factor that is secreted by hMSCs. Moreover, IL-10 secretion by MSCs is critical for their immunomodulatory functions in immune disease models, including those for arthritis [28].

In this study, we investigated if treatment with an HMA in combination with an HDACi could increase the gene expression of IL-10 and IDO by MSCs, based on the success of our previous approach to upregulate key differentiation factors in MSCs [22]. The enhanced IL-10 and IDO expression in epi-hMSCs after combination treatment confirmed that epigenetic modification can modulate gene expression levels. The epi-hMSCs also had greater immunosuppressive effects on T-cell proliferation and cytokine expression, and Th17 cell differentiation, than the untreated hMSCs.

Th17 cells protect the host against extracellular pathogens that are encountered at mucosal surfaces. They also play a detrimental role in experimental murine models of inflammatory diseases, such as multiple sclerosis and RA, as well as in human inflammatory bowel disease and psoriasis [29–32]*.* A previous study demonstrated that MSCs inhibit human Th17 cell differentiation and function [33]. IL-2 supports the proliferation [34–37] and survival [38] of T cells, as well as the differentiation of naive T cells into effector and memory cells, including Th17 cells [39–42]. In our study, coculture with epi-hMSCs suppressed the production of IL-2 compared with its expression in the cultures under Th17 conditions alone or with untreated hMSCs.

Effector T cells, including Th17 cells, may differ in patients with RA and healthy individuals due to the continuous stimulation and attempts at immunosuppression in the setting of autoimmunity [43]. Importantly, coculture with epi-hMSCs, as opposed to no or untreated hMSCs, resulted in lower Th17 cytokine secretion and proliferation by cells from patients with RA. These findings support the potential of epi-hMSCs for the treatment of RA.

Although the results of this study on epi-hMSCs are promising, they are limited by the fact that we did not demonstrate such effects in in-vivo models. However, as effective regulation of Th17 immune responses was observed during proliferation and differentiation of Th17 cells and cytokine secretion, the results suggest that epigenetic modification of MSCs deserves further study.

## Conclusions

We found that treatment with the combination of an HMA and an HDACi increased the immunomodulatory properties of hMSCs. Our results support the approach of enhancing the function of hMSCs via epigenetic modification. Further studies on the safety of epi-hMSCs are required prior to their use as therapeutics in RA and related diseases. In addition, future research should focus on the development of novel epigenetic markers to select optimal hMSCs and methodologies to increase the therapeutic effects of epi-hMSCs.

## Abbreviations

5-AZA: 5-Azacitidine
A2V10: 2 μM 5-azacitidine + 10 mM valproic acid
A2V5: 2 μM 5-azacitidine + 5 mM valproic acid
BM: Bone marrow
CFSE: Carboxyfluorescein succinimidyl ester
D100T100: 100 nM 5-aza-2′-deoxycytidine + 100 nM trichostatin A
D100T500: 100 nM 5-aza-2′-deoxycytidine + 500 nM trichostatin A
DEC: 5-Aza-2′-deoxycytidine
ELISA: Enzyme-linked immunosorbent assay
epi-hMSC: Epigenetically modified human mesenchymal stromal cell
FCS: Fetal calf serum
HDACi: Histone deacetylation inhibitor
HMA: Hypomethylating agent
hMSC: Human mesenchymal stromal cell
IDO: Indoleamine 2,3-dioxygenase
IFN-γ: Interferon gamma
IL: Interleukin
MACS: Magnetic-activated cell sorting
MSC: Mesenchymal stromal cell
PBMC: Peripheral blood mononuclear cell
PCR: Polymerase chain reaction
RA: Rheumatoid arthritis
SD: Standard deviation
SFMC: Synovial fluid mononuclear cell
Th: T-helper
TNF: Tumor necrosis factor
TSA: Trichostatin A
VPA: Valproic acid
